# *HIF1A* (rs11549465) and* AKNA* (rs10817595) Gene Polymorphisms Are Associated with Primary Sjögren's Syndrome

**DOI:** 10.1155/2017/5845849

**Published:** 2017-04-06

**Authors:** Gabriela Hernández-Molina, José Manuel Rodríguez-Pérez, Javier Fernández-Torres, Guadalupe Lima, Nonanzit Pérez-Hernández, Alberto López-Reyes, Gabriela Angélica Martínez-Nava

**Affiliations:** ^1^Department of Immunology and Rheumatology, Instituto Nacional de Ciencias Médicas y Nutrición Salvador Zubirán, Mexico City, Mexico; ^2^Department of Molecular Biology, Instituto Nacional de Cardiología Ignacio Chávez, Mexico City, Mexico; ^3^Synovial Fluid Laboratory, Instituto Nacional de Rehabilitación Luis Guillermo Ibarra Ibarra, Mexico City, Mexico; ^4^Biological and Health Sciences PhD Program, Universidad Autónoma Metropolitana Iztapalapa, Mexico City, Mexico

## Abstract

*Objective*. To evaluate the allele and genotype frequencies of polymorphic sites of* HIF1A* and* ANKA* genes in primary Sjögren's syndrome (pSS).* Methods*. We included 110 patients with pSS and 141 ethnically matched healthy controls. Three* HIF1A* gene polymorphisms* (Pro582Ser*,* Ala588Thr*, and* C191T)* and two* AKNA gene* polymorphisms* (−1372C>A* and* Pro624Leu)* were genotyped using TaqMan probes in a Real-Time PCR instrument. Associations between pSS and genotypes, alleles, and inheritance models of the SNPs of interest were evaluated by logistic regression adjusted by age and gender.* Results*. The *C*/*T* genotype and the *T* allele of the* HIF1A Pro582Ser* polymorphism protected against pSS (OR = 0.22; 95% CI = 0.09–0.52; *P* < 0.01; OR = 0.26; 95% CI = 0.12–0.58; *P* < 0.01, resp.), whereas under a recessive model adjusted by age and gender, the* AKNA −1372C>A* polymorphism A/A genotype was associated with an increased risk of pSS (OR = 2.60; 95% CI = 1.11–6.12; *P* = 0.03).* Conclusions*. We identified* HIF1A Pro582Ser T* allele and *C*/*T* genotype as well as* AKNA −1372C>A* polymorphism A/A genotype as genetic factors associated with pSS. Further studies in other populations are needed to validate our findings and research is warranted in order to shed some light on their functional implications across biological pathways in this disease.

## 1. Introduction

Primary Sjögren's syndrome (pSS) is a chronic autoimmune disorder characterized by diminished lacrimal and salivary gland secretion, resulting in keratoconjunctivitis sicca and xerostomia [1]. Patients with pSS mount a localized immunological response against the epithelial component of exocrine glands which produces a destructive inflammatory infiltrate comprised of activated T and B cells, which ultimately lead to a progressive exocrine function lost [2]. Epidemiological and genetic studies support the idea of a complex etiology of the disease, involving variants in numerous genes with functional implications across multiple biological pathways, including inflammation and oxidative burst [3, 4].

In these pathways, one of the key molecules is the hypoxia inducible factor-1*α* (HIF-1*α*), which is the main transcriptional regulator of cellular and developmental response to hypoxia. The deregulation and overexpression as well as genetics variants of this gene have been related to angiogenesis, cell proliferation, metabolism, cancer biology, and osteoarthritis [5, 6]. Although the importance of HIF-1*α* is well described in the context of hypoxia, recent data suggest that it may also play a role in innate immunity. HIF-1*α* is also a key player in the integration of the T cell receptor (TCR) and cytokine receptor-mediated signals of CD4+ helper T cells [7]. In addition, HIF-1*α* enhances Th17 development through direct transcriptional activation of RAR-related orphan receptor gamma t (ROR*γ*t) [8]. This subpopulation is highly increased in salivary gland tissue of patients with Sjögren's syndrome (SS) and in a mouse model of SS [9]. Conversely, in a HIF-1*α* conditional knockout mouse model with dry-eye, the lacrimal glands exhibited increased acinar cell apoptosis and loss of glandular structures and polarities and, on the other hand, the blockage of HIF-1*α* COX-2 axis enhanced inflammation [10]. Furthermore, the participation of HIF-1*α* has been studied in inflammatory and autoimmune disorders. There is evidence that points out the key role of HIF-1*α* in the regulation of several pathophysiological processes such as rheumatoid arthritis (RA) and systemic lupus erythematous, especially producing synovial inflammation as well as glomerular and interstitial inflammation [11, 12].

Another transcription factor involved in the immune response is the AT-hook transcription factor (AKNA). This protein targets genes with A/T-rich promoters and regulates specific genes expression involved in the establishment of the immune response. Particularly, AKNA upregulates the expression of two costimulatory molecules, CD40 and CD40L, in T and B-lymphocytes, as well as natural killer and dendritic cells [13]. To date, AKNA gene polymorphisms as well as its expression had been associated with other human pathologies in which the immune system plays an important role such as cervical cancer [14], leukemia [15], Vogt-Koyanagi-Harada's syndrome [16], and hepatitis C virus infection [17].

The aforementioned evidence suggests the involvement of* HIF1A* and* AKNA* genes in immune system pathologies. In this context, identifying genetic variants of these two genes in pSS will allow a more precise definition of pathogenic mechanisms of the disease.

The aim of this study was to explore the role of* HIF1A* and* AKNA* gene polymorphisms and their association with pSS in a well characterized-clinical cohort of Mexican patients. We also analyzed the association of glandular, extraglandular, and serological features among pSS patients with these genes polymorphisms that codify transcriptional regulator factors expressed in T and B cells.

## 2. Materials and Methods

### 2.1. Subjects

We included 110 consecutive Mexican mestizo patients with the diagnosis of pSS according to the American-European Consensus Group SS classification criteria [18], who regularly attended a tertiary referral care center at Mexico City. Patient's clinical records were carefully reviewed according to a preestablished protocol. We registered demographic, glandular features (oral and ocular symptoms, parotid enlargement, Schirmer-I test, nonstimulated whole salivary flow (NSWSF), and fluorescein staining) and extraglandular features (nonerosive arthritis, neurological involvement [polyneuropathy, mononeuropathy, cranial nerves involvement, dysmyelinization, disautonomy radiographic or electrophysiology diagnosed, or biopsy proven], skin vasculitis [palpable purpura or biopsy proven], and Raynaud's phenomenon), as well as serological variables (anti-Ro/SSA and anti-La/SSB antibodies). We excluded patients with another concomitant autoimmune disease.

We included 141 healthy volunteers from a referral orthopedic center at the same city where the patients were recruited. Although they did not have complete evaluation to rule out the presence of pSS, they did not complain of oral/ocular symptoms and did not self-report any autoimmune condition.

All participants were not related and self-reported Mexican mestizo ancestry (at least three generations including their own).

### 2.2. Genetic Analysis

Genomic DNA was extracted from leucocytes, after collecting a peripheral blood sample using the Lahiri and Numberger method [19]. We evaluate three codifying polymorphisms of* HIF1A* gene, two missense (*Pro582Ser*, rs11549465 and* Ala588Thr,* rs11549467) and one 3′ UTR (*C191T*, rs2057482) and two* AKNA* gene polymorphisms, one in the promoter region (−1372*C>A*, rs10817595) and other in the coding region (*Pro624Leu*, rs3748176).

The SNPs were genotyped using 5′ exonuclease TaqMan genotyping assays on a StepOne Plus Real-Time PCR system (Applied Biosystems, Foster City, USA). The results were analyzed in allelic discrimination software StepOnePlus v2.3. Previously sequenced samples of the five polymorphisms of all different genotypes were included as positive controls and the results were found to be consistent.

### 2.3. Statistical Analysis

Hardy-Weinberg equilibrium (HWE) was calculated for each polymorphism using Fisher's exact test. According to the variable type, patients and controls were compared using Mann–Whitney* U* test or Fisher's exact test when appropriate. Allelic and genotypic frequencies were estimated for all polymorphisms (rs11549465, rs11549467, and rs2057482 of* HIF1A* gene; rs10817595 and rs3748176 of* AKNA* gene) and inheritance models were constructed and assessed for the polymorphisms whose frequencies allowed it.

The associations between pSS, genotypes, alleles, and inheritance models of the SNPs of interest were evaluated by logistic regression models adjusted by age and gender. In order to reject a possible type I error, bootstrap-resampling analysis (100 replications) was performed. All the statistical analyses and graphs were performed using the statistical package STATA v12.1. (StataCorp, Texas, USA) and considering a significance level with *α* = 0.05.

### 2.4. Ethical Considerations

The study complies with the Declaration of Helsinki and was approved by the Institutional Bioethics and Research Committee of the Instituto Nacional de Ciencias Médicas y Nutrición. All participants signed an informed consent letter.

## 3. Results

### 3.1. Characteristics of the Study Population

Most of the patients were females (95.4%), with a median age of 55 years and median disease duration of 7.8 years.  Table 1 depicts their clinical and serological features. In contrast, in the control group most of the subjects were males (30.8% versus 4.5% pSS, *P* < 0.01) and younger than the pSS group (41 versus 55 y/o, resp., *P* < 0.01).

### 3.2. Allele and Genotype Frequencies

Allele and genotype frequencies of polymorphic sites in patients with pSS and controls are depicted in Table 2. Observed and expected frequencies of all polymorphisms were in HWE in the total sample size. The genotypic frequencies of* HIF1A Pro582Ser* (rs11549465) and* AKNA −1372C>A* (rs10817595) polymorphisms were not in HWE in the control group (*P* = 0.04  and  *P* = 0.01, resp.); nevertheless after Bonferroni correction for multiple comparisons the observed and expected genotypic frequencies given HWE for both polymorphisms were not statistically different in this group (Bonferroni corrected *α* = 0.003).

A similar distribution of* HIF1A* (rs11549467 and rs207482) as well as* AKNA* (rs3748176) polymorphisms was observed. In contrast, a lower frequency of the* C/T* genotype and the* T* allele of the* HIF1A* (rs11549465) polymorphism was found among patients with pSS. In addition in the inheritance model, we found a higher frequency of the* A/A* genotype of the* AKNA* (rs10817595) gene polymorphism in pSS (Table 2).

In the logistic regression adjusted by age and gender, the* C/T* genotype and the* T* allele of the* HIF1A* (rs11549465) polymorphism protected against pSS (OR = 0.22; 95% CI = 0.09–0.52; *P* < 0.01; OR = 0.26; 95% CI = 0.12–0.58; *P* < 0.01, resp.) (Table 3).

On the other hand, under a recessive inheritance model adjusted by age and gender, the* AKNA* (rs10817595)* A/A* genotype was associated with increased risk of pSS (OR = 2.60; 95% CI = 1.11–6.12; *P* = 0.03) (Table 3).

We explored the association of glandular, extraglandular, and serological features among pSS patients and the associated* HIF1A* and* AKNA* gene polymorphisms (data not shown). Parotid enlargement was the only feature that was associated with the* HIF1A* gene polymorphism (rs11549465), conferring protection for the* C/T* genotype versus* C/C* (OR = 0.22; 95% CI 0.05–0.90; *P* = 0.03). In addition, the allele* T* of the same polymorphism (rs11549465) also conferred protection when compared with the* C* allele (OR = 0.24; 95% CI = 0.65–0.95; *P* = 0.04). We did not find any other association with the rest of the* HIF1A or AKNA* gene polymorphisms tested.

## 4. Discussion

The pathogenesis of Sjögren's syndrome likely involves complex interactions between genes and environment; thus the study of genetic factors is an important area of research. For instance, genetic variation may influence molecular processes such as splicing and posttranslational modifications [3]. Herein, we report the allele and genotype frequencies of polymorphic sites of* HIF1A* and* ANKA* genes in pSS patients and their association with the susceptibility to this disease.

HIF-1*α* regulates the expression of over 100 genes that control the major cellular functions including apoptosis, cell proliferation, glucose metabolism, erythropoiesis, iron metabolism, and angiogenesis. It is a master regulator of the cellular response to hypoxia and is widely expressed in all immune system cells.* HIF1A* expression can be triggered not only by hypoxia, but also by diverse stimuli associated with leukocyte activation and inflammation [20, 21]. In this sense, several inflammatory cytokines, such as IL-1, TNF-*α*, IL-4, and TGF-*β*, have been shown to induce HIF-1*α* in multiple cell types during normoxic conditions. Induced HIF-1*α* may specifically play a role in the innate immune response to viral infection; however it is still unknown if there is a relationship with type I IFN (IFN-*α* or IFN-*β*) and type II IFN (IFN-*γ*) [22].

On the other hand, there is evidence showing that HIF-1*α* upregulates the expression of inflammatory cytokines including IFN-*γ* in synovial fibroblast of RA patients [23]. In this sense, IFN*γ* has been associated with more severe forms of SS, characterized by high grade infiltration of lymphocytes, macrophages, and dendritic cells as well as with gastric NHL lymphomas [24].

HIF-1*α* also participates in the integration of the TCR and cytokine receptor-mediated signals of CD4+ helper lineage (e.g., the activation of P13 kinase/mTOR pathway) as well as in CD8+ effectors differentiation, in a fashion independent of oxygen availability [7]. HIF-1*α* enhances Th17 development and also attenuates Treg development by binding to transcription factor Foxp3 and targeting it for proteasomal degradation [8], although paradoxically it is also required for optimal Treg function in disease models [25]. HIF-1*α* is also essential for normal B-cell development and self-tolerance in a chimeric HIF-1*α*^−/−^⇒Rag2^−/−^ murine model [26].

In RA, HIF-1*α* induces both angiogenesis and proinflammatory mechanisms [27], and in lupus nephritis larger amounts of HIF-1*α* in both glomerular and tubulointerstitial areas evaluated by immunohistochemical staining have been observed [28].

Thus, there is significant evidence that the HIF-1*α* pathway is important to several aspects of innate and adaptive immune function, differentiation, and cytokine expression. Herein we identify that the* HIF1A Pro582Ser* (rs11549465)* T* allele and* C/T* genotype were protective for pSS. Interestingly, in a HIF-1*α* conditional* knockout* mouse model with dry-eye, the lacrimal glands exhibited increased acinar cell apoptosis and loss of glandular structures and polarities, and the blockage of HIF-1*α* COX-2 axis enhanced inflammation [10]. This evidence suggests a potential protective effect of* Pro568Ser HIF1A* polymorphism in pSS.

HIF1A* Pro582Ser* (rs11549465) polymorphism is located in the 12th exon of the gene and confers a substitution of a proline to a serine in the 582-amino acid residue of the codified protein. This polymorphism fell in the oxygen-dependent degradation domain of HIF-1*α* and in one of its transactivation domains suggesting its participation in HIF-1*α* function [29]. Previously in vitro studies have showed that the carriers of these polymorphic variants confer greater stability and promote an elevated transcriptional activity of HIF-1*α* when compared to the wild type, under normoxic and hypoxic conditions [30, 31].

These works point out the relevance and functionality of this polymorphism over the transcription of HIF-1*α* target genes that are involved in pSS pathology.

Previous genetic studies in pSS rarely reported association of candidate genes with clinical features. For instance, a trend for association between genetic variation in SNP of* SLC25A40* and* PKN1* and fatigue has been reported [32]. Herein we observed that* HIF1A Pro582Ser* (rs11549465)* T* allele and* C/T* genotype protected against parotid enlargement among the pSS group.

On the other hand, AKNA is an AT-hook transcription factor, which upregulates the expression of costimulatory cell surface molecules, such as CD40 and CD154, on immune response cells [33]. These costimulatory molecules are critical for the triggering of the humoral response in B cells and priming of T lymphocytes [34]. In pSS patients, the concentration of CD154 in serum and the expression of CD154 in T CD4+ cells are significantly higher compared with healthy controls [35, 36]. Interestingly, the overexpression of CD154 reported by Belkhir et al. [37] was not due to a difference in DNA methylation patterns. This points out the relevance of AKNA as transcription regulator of CD154 and CD40 in pSS pathology.

Herein we found that the* AKNA −1372C>A* (rs10817595)* A/A* genotype under a recessive inheritance model was associated with an increased risk of pSS. In contrast, loss of AKNA in a murine model results in mice that die suddenly at 10 days of life with diffuse inflammatory lesions predominantly in the lungs, induced mainly by IL-1-*β* and IFN-*γ* and development of anti-DNA autoantibodies [38].

The polymorphism associated with higher risk of pSS in the present work (*−1372C>A*, rs10817595) is located in the promoter region of* AKNA* gene. This polymorphism has been proposed as an expression quantitative trait loci (eQTL), given that in peripheral blood mononuclear cells the *A* allele promotes higher expression level of* AKNA* [14].

To date, the molecular mechanism involved in* HIF1A* and* AKNA* expression is still a matter of research. For instance,* HIF1A* can extend different and sometimes opposing roles and this is probably sustained by their unusual capacity to generate multiple transcripts and proteins. In addition, the apparent conflicting results in knockout-deletion mice models for both genes should be taken with caution regarding overreliance on these models in isolation.

Genome-wide association studies (GWAS) are an important tool for identifying disease susceptibility genes. The existing GWAS have not identified* HIF1A* and* AKNA* as genes associated with the disease [4, 39]. Considering that the majority of these GWAS have been done in Caucasian or European populations, the results do not necessarily provide information for the Mexican population. In addition, the genetic risk varies across ethnicity and genetic heterogeneity is a well-recognized reason for failure to replicate genetic association findings. In fact a recent GWAS study among European and Asian SS patients showed striking differences between both populations, with high heterogeneity especially in the MHC and representative SNPs. Thus genetic associations with SS differ markedly by ancestry [40]. Moreover, it is known that GWAS have limitations to detect low frequency and small magnitude variants associated with the disease in question.

A possible limitation of this study is the fact that we observed Hardy-Weinberg disequilibrium (HWD) in the control group for* AKNA −1372C>A* (rs10817595) and* HIF1A Pro582Ser* (rs11549465) polymorphisms. It is highly unlikely that the HWD found was due to genotyping errors because in each assay we had previously genotyped positive controls, and we had 100% of concordance in each performed assay. Besides, taking into account the Bonferroni correction for multiple comparisons the *P* values for these HWE tests were highly above the significance level. Furthermore, the allele frequencies observed for the polymorphism in which the HWE *P* value was the lowest (*AKNA* rs10817595) are consistent with that reported in the 1000 genomes project for Mexicans (41% for the *A* allele and 59% for the *C* allele) [41].

In addition as pSS is a disease that primary affects women between ages 30 and 50 and our controls were predominantly men and young, we adjusted our results by gender and age in order to minimize any possible influence that these variables could have over the associations found.

In conclusion, we identified* HIF1A Pro582Ser* (rs11549465)* T* allele and* C/T* genotype as well as* AKNA −1372C>A* (rs10817595)* A/A* genotype as susceptibility genetic factors of pSS, conferring the former a decreased and the latter an increased risk of pSS among Mexican mestizo population. Notwithstanding, as these individual polymorphisms only confer a small risk change, further studies in other populations are needed to validate our findings and to assess their participation in additive models with other known risk alleles as well as with their interaction with epigenetic and environmental factors. Therefore, research is warranted in order to shed some light on their functional implications across biological pathways in this disease.

## Figures and Tables

**Table 1:** Clinical and serological characteristics of PSS patients.

Variable	Primary Sjögren's syndrome (*n* = 110)
Median age in years (interquartile range)	55 (17)
Female, *n* (%)	105 (95.45)
Median disease duration in years (interquartile range)	7.8 (10)
Ocular symptoms, *n* (%)	90 (90.9)
Oral symptoms, *n* (%)	103 (93.6)
Parotid enlargement, *n* (%)	60 (54.6)
Positive Schirmer-I test, *n* (%)	96 (87.3)
Impaired nonstimulated whole salivary flow, *n* (%)	96/103 (93.2)
Positive fluorescein staining, *n* (%)	53/80 (66.2)
Positive anti-Ro/SSA antibodies, *n* (%)	91 (82.7)
Positive anti-La/SSB antibodies, *n* (%)	53 (48.18)
Skin vasculitis, *n* (%)	2 (1.8)
Neurological involvement, *n* (%)	12 (10.9)
Raynaud's phenomenon *n* (%)	11 (10)
Nonerosive arthritis, *n* (%)	23 (20.9)

**Table 2:** Genotype and allele frequency by study group of HIF1A and AKNA polymorphisms.

Polymorphism	Frequency *n* (%)			*P* value^c^
	Total study population (*n* = 251)	Controls (*n* = 141)	Primary Sjögren's syndrome (*n* = 110)	
*HIF1A Pro582Ser (rs11549465)*				
C/C	188 (78.0)	94 (69.6)	94 (88.7)	<0.01
C/T	53 (22.0)	41 (30.4)	12 (11.3)	
T/T	0	0	0	
Alleles				
C	429 (89.0)	229 (84.8)	200 (94.3)	<0.01
T	53 (11.0)	41 (15.2)	12 (5.70)	
HWE^a^ *P* value	0.09	0.04	0.99	
*HIF1A Ala588Thr (rs11549467)*				
G/G	198 (99.5)	90 (98.9)	108 (100.0)	0.46
G/A	1 (0.5)	1 (1.10)	0	
A/A	0	0	0	
Alleles				
G	397 (99.8)	181 (99.4)	216 (100.0)	0.46
A	1 (0.25)	1 (0.60)	0	
HWE *P* value	0.99	0.99	—	
*HIF1A C191T (rs2057482)*				
T/T	190 (83.0)	96 (80.7)	94 (85.4)	0.38
T/C	39 (17.0)	23 (19.3)	16 (14.6)	
C/C	0	0	0	
Alleles				
T	419 (91.5)	215 (90.3)	204 (92.7)	0.40
C	39 (8.52)	23 (9.7)	16 (7.27)	
HWE *P* value	0.38	0.60	0.99	
*AKNA −1372 C>A (rs10817595)*				
C/C	72 (29.2)	41 (29.1)	31 (29.2)	0.11
C/A	137 (55.5)	84 (59.6)	53 (50.0)	
A/A	38 (15.4)	16 (11.3)	22 (20.8)	
Inheritance models				
C/A + A/A	175 (70.85)	100 (70.9)	75 (70.8)	0.54
A/A^b^	38 (15.4)	16 (11.3)	22 (20.8)	0.03
Alleles				
C	281 (56.9)	166 (58.9)	115 (54.2)	0.18
A	213 (43.1)	116 (41.1)	97 (45.8)	
HWE *P* value	0.05	0.01	0.99	
*AKNA Pro624Leu (rs3748176)*				
G/G	69 (28.1)	39 (28.1)	30 (28.3)	0.56
G/A	132 (53.9)	78 (56.1)	54 (50.9)	
A/A	44 (18.0)	22 (20.8)	22 (20.8)	
Inheritance models				
G/A + A/A	176 (71.8)	100 (71.9)	76 (71.7)	0.54
A/A^b^	44 (18.0)	22 (20.8)	22 (20.8)	0.20
Alleles				
G	270 (55.1)	156 (56.1)	114 (53.8)	0.34
A	220 (44.9)	122 (43.9)	98 (46.2)	
HWE *P* value	0.20	0.12	0.85	

^a^HWE: Hardy Weinberg Equilibrium.

^b^Recessive inheritance model; the reference group is formed by C/A and C/C genotype carriers or G/A and G/G genotype carriers for each AKNA polymorphism, respectively.

^c^Fisher's exact test *P* value.

**Table 3:** Odds ratio for primary Sjögren's syndrome by polymorphisms genotypes, alleles, and inheritance models.

Polymorphism	*N* (controls/cases)	OR^a^ (95% CI)	*P* value
*HIF1A Pro582Ser (rs11549465)*			
C/C	94/94	1.00	
C/T	41/12	0.22 (0.09–0.52)^c^	<0.01
T/T	0/0	—	—
Alleles			
C	229/200	1.00	
T	41/12	0.26 (0.12–0.58)^c^	<0.01
*HIF1A C191T (rs2057482)*			
T/T	96/94	1.00	
T/C	23/16	0.43 (0.18–1.04)	0.06
C/C	0/0	—	—
Alleles			
T	215/204	1.00	
C	23/16	0.47 (0.21–1.07)	0.07
*AKNA −1372 C>A (rs10817595)*			
C/C	41/31	1.00	
C/A	84/53	0.64 (0.31–1.32)	0.23
A/A	16/22	1.94 (0.73–5.15)	0.19
Inheritance models			
C/A + A/A	100/75	0.82 (0.41–1.64)	0.58
A/A^b^	16/22	2.60 (1.11–6.12)^c^	0.03
Alleles			
C	166/115	1.00	
A	116/97	1.20 (0.78–1.86)	0.42
*AKNA Pro624Leu (rs3748176)*			
G/G	39/30	1.00	
G/A	78/54	0.76 (0.37–1.58)	0.46
A/A	22/22	1.56 (0.62–3.96)	0.35
Inheritance models			
G/A + A/A	100/76	0.92 (0.46–1.83)	0.80
A/A^b^	22/22	1.88 (0.85–4.16)	0.12
Alleles			
G	156/114	1.00	
A	122/98	1.17 (0.75–1.81)	0.48

^a^Odds ratio adjusted by age and gender.

^b^Recessive inheritance model; the reference group is formed by C/A and C/C genotype carriers or G/A and G/G genotype carriers for each AKNA polymorphism, respectively.

^c^Odds ratio statistically significant after bootstrap resampling (100 repetitions).
